# Gut Bacteria Mediate Nutrient Availability in *Drosophila* Diets

**DOI:** 10.1128/AEM.01401-20

**Published:** 2020-12-17

**Authors:** Danielle N. A. Lesperance, Nichole A. Broderick

**Affiliations:** a Department of Molecular and Cell Biology, University of Connecticut, Storrs, Connecticut, USA; b Institute for Systems Genomics, University of Connecticut, Storrs, Connecticut, USA; University of Manchester

**Keywords:** *Drosophila melanogaster*, microbiome, host-microbe interactions, nutritional physiology, protein, carbohydrates, moisture, tryptophan

## Abstract

Drosophila melanogaster gut microbes play important roles in host nutritional physiology. However, these associations are often indirect, and studies typically are in the context of specialized nutritional conditions, making it difficult to discern how microbiome-mediated impacts translate to physiologically relevant conditions, in the laboratory or nature. In this study, we quantified changes in dietary nutrients due to D. melanogaster gut bacteria on three artificial diets and a natural diet of grapes. We show that under all four diet conditions, bacteria altered the protein, carbohydrates, and moisture of the food substrate. An in-depth analysis of one diet revealed that bacteria also increased the levels of tryptophan, an essential amino acid encountered scarcely in nature. These nutrient changes result in an increased protein-to-carbohydrate (P:C) ratio in all diets, which we hypothesized to be a significant determinant of microbiome-mediated host nutritional physiology. To test this, we compared life history traits of axenic flies reared on the three artificial diets with increased P:C ratios or continuous bacterial inoculation. We found that while on some diets, an environment of nutritional plenitude had impacts on life history, it did not fully explain all microbiome-associated phenotypes. This suggests that other factors, such as micronutrients and feeding behavior, likely also contribute to life history traits in a diet-dependent manner. Thus, while some bacterial impacts on nutrition occur across diets, others are dictated by unique dietary environments, highlighting the importance of diet-microbiome interactions in D. melanogaster nutritional physiology.

**IMPORTANCE** Both in the laboratory and in nature, D. melanogaster-associated microbes serve as nutritional effectors, either through the production of metabolites or as direct sources of protein biomass. The relationship between the microbiome and the resulting host nutritional physiology is significantly impacted by diet composition, yet studies involving D. melanogaster are performed using a wide range of artificial diets, making it difficult to discern which aspects of host-microbe interactions may be universal or diet dependent. In this study, we utilized three standard D. melanogaster diets and a natural grape diet to form a comprehensive understanding of the quantifiable nutritional changes mediated by the host microbial community. We then altered these artificial diets based on the observed microbe-mediated changes to demonstrate their potential to influence host physiology, allowing us to identify nutritional factors whose effects were either universal for the three artificial diets or dependent on host diet composition.

## Abstract (precis)

Both in the laboratory and in nature, D. melanogaster-associated microbes serve as nutritional effectors, either through the production of metabolites or as direct sources of protein biomass. The relationship between the microbiome and the resulting host nutritional physiology is significantly impacted by diet composition, yet studies involving D. melanogaster are performed using a wide range of artificial diets, making it difficult to discern which aspects of host-microbe interactions may be universal or diet dependent. In this study, we utilized three standard D. melanogaster diets and a natural grape diet to form a comprehensive understanding of the quantifiable nutritional changes mediated by the host microbial community. We then altered these artificial diets based on the observed microbe-mediated changes to demonstrate their potential to influence host physiology, allowing us to identify nutritional factors whose effects were either universal for the three artificial diets or dependent on host diet composition.

## INTRODUCTION

Gut microbes have important functions in host development, immunity, intestinal homeostasis, and metabolism across model organisms (1–4). There is growing appreciation for the gut microbiome’s role in host nutritional intake, with effects through both the microbial catabolism of nutrients and the biosynthesis of metabolites (5–7). The use of the Drosophila melanogaster model has revealed significant roles for gut microbes in activating nutritional signaling pathways (8, 9), stimulating protein nutrition (10, 11), and catabolizing dietary carbohydrates (12). A small number of microbe-derived metabolites, including acetate and B vitamins, have been identified that promote D. melanogaster nutrition either directly or via impacts on feeding behavior (13, 14). Interactions between host and microbiome are strongly influenced by fly diet. In particular, under conditions of nutrient scarcity, gut bacteria decrease development time and increase life span (8–11). On the other hand, excess dietary protein is thought to diminish the impact of the microbiome (specifically bacteria) on development and life span (15).

Despite the many benefits of using D. melanogaster to investigate a spectrum of biological processes, the range of diets used for fly maintenance across the field can complicate interpretation (16, 17). Recently, we showed that diet compositions between studies varied greatly in both the amounts and types of components used (i.e., inactive yeast versus brewer’s yeast versus yeast extract), which ultimately resulted in diets containing a wider-than-appreciated range of macro- and micronutrients (17). Many diets are reported as “standard” in the literature as well, often without basis (for example, the concentration of protein in so-called standard diets analyzed in our study ranged from 6.33 to 77.93 g per liter), making the interpretation of differences in nutritional environments and, as a result, nutrition-mediated phenotypes difficult (17). These discrepancies make it challenging to contextualize studies within and across fields of interest, particularly in the case of nutrition and the microbiome, which depend so heavily on dietary composition.

In this study, we investigate microbial impacts on D. melanogaster nutrition by analyzing the nutritional contents of three standard fly diets with and without bacterial inoculation. Two of the diets that we tested, the Bloomington standard and Bloomington cornmeal-molasses-yeast (CMY) diets, are broadly used or are at least the basis of diets used by the D. melanogaster community in a range of fly research areas. Thus, they provide a general understanding of microbe-diet relationships in diets in use by a broad swath of the community, in contrast to more specialized or defined diets, whose physiological implications may be difficult to interpret. We assess microbial impacts on fly life history and identify microbe-mediated changes in protein, carbohydrate, and moisture contents, as well as the amino acid tryptophan, as potential mechanisms of microbial modulation of host physiology. We go on to assess nutritional changes due to bacteria on a natural fly diet of grapes to contextualize the overall relevance of our findings.

## RESULTS

### *Drosophila* gut bacteria impact the nutrient content of fly food.

**FIG 1:**
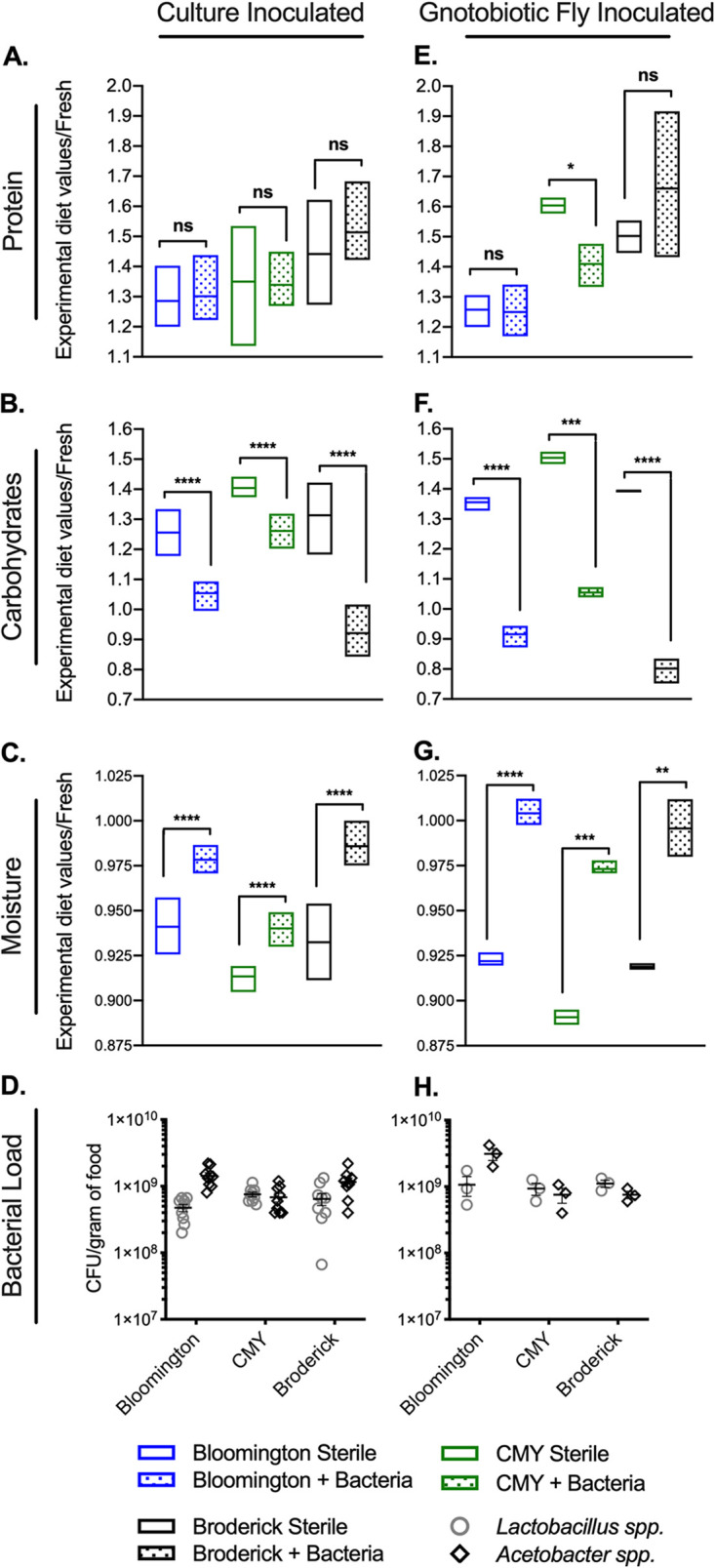
*Drosophila* gut bacteria impact the nutrient content of fly food. (A to D) Protein (A), carbohydrates (B), moisture (C), and bacterial load (D) in Bloomington, CMY, and Broderick diets 14 days after direct inoculation with PBS (Sterile) or 10^4^ cells of a 4-species bacterial cocktail (+Bacteria). (E to H) Protein (E), carbohydrates (F), moisture (G), and bacterial load (H) in Bloomington, CMY, and Broderick diets 14 days after inoculation via axenic flies (Sterile) or gnotobiotic flies previously fed 10^4^ cells of a 4-species bacterial cocktail (+Bacteria) (see Fig. S1 in the supplemental material for bacterial counts within gnotobiotic flies at the time of diet inoculation). Nutritional data (A to C and E to G) are expressed as raw values from each nutritional test in treated samples divided by raw values for fresh, untreated diets (see Fig. S1 in the supplemental material for raw nutritional data). Bars represent minimum and maximum values and means from 9 (A to D) or 3 (E to H) biological replicates. Statistical differences between sterile and +Bacteria treatments within each diet were determined using unpaired two-tailed *t* tests. Bacterial loads (D and H) are expressed as CFU per gram of food, with each point representing an individual replicate. Lines and error bars represent means ± standard errors of the means (SEM). No statistical differences between *Lactobacillus* spp. and *Acetobacter* spp. within each diet and between diets were detected via two-way ANOVA. Bacterial growth was not detected in sterile treatments and is not shown. Significance is expressed as follows: ns, not significant (*P* > 0.05); *, *P* ≤ 0.05; **, *P* ≤ 0.01; ***, *P* ≤ 0.001; ****, *P* ≤ 0.0001.

To address how the microbiome interacts with diet under laboratory conditions, we prepared three standard diets: the Bloomington standard diet, the Bloomington CMY diet, and our laboratory standard diet, called the Broderick standard diet here. We inoculated bottles of sterile food with either phosphate-buffered saline (PBS) or a bacterial cocktail of four common D. melanogaster gut microbes, Lactobacillus plantarum, Lactobacillus brevis, Acetobacter pasteurianus, and Acetobacter tropicalis (+Bacteria), in equal proportions. Treated food was incubated at 25°C for 14 days to simulate the length of time that bacteria would associate with food over the course of a typical fly life cycle (from egg laying to adult eclosion), although larval churning of food was not simulated. After incubation, food was collected and analyzed for bacterial load and nutritional content, including protein, carbohydrates, ash, fat, and moisture. Across the three diets, bacterial treatment decreased carbohydrates and increased moisture compared to PBS-treated controls, with generally no significant change in protein, ash, or fat levels (Fig. 1A to C; see also Fig. S1A in the supplemental material). To determine if this relationship between microbes and fly food persisted under a natural method of inoculation, including effects of larval mixing, we generated gnotobiotic flies by feeding axenic adults the same 4-species bacterial cocktail and then placed either axenic flies (sterile treatment) or gnotobiotic flies (+Bacteria treatment) on sterile food. After 4 days, all flies were removed, and food was further incubated for 10 days to allow larvae to pupate and reduce the confounding effects of the host biomass on nutritional analysis. As food was collected for sampling, emerged adults, larvae, and pupae were discarded. The nutritional values were generally consistent with the levels observed in food directly inoculated with bacterial cultures, although the degrees of carbohydrate reduction and moisture increase were greater for gnotobiotic inoculation than for culture inoculation (Fig. 1E to G; Fig. S1B) despite the genus-level bacterial loads being similar for each inoculation method and across diets (Fig. 1D and H). While protein levels appeared to decrease in the CMY +Bacteria treatments, we noted an associated increase in protein in the sterile CMY treatment under gnotobiotic conditions compared to culture inoculation conditions. Thus, we attribute the difference in protein between sterile and +Bacteria CMY treatments to possible “contamination” by host tissues, which artificially inflated the sterile treatment protein levels, since the protein levels in the +Bacteria treatments were not different between gnotobiotic and culture inoculation methods.

**FIG 2:**
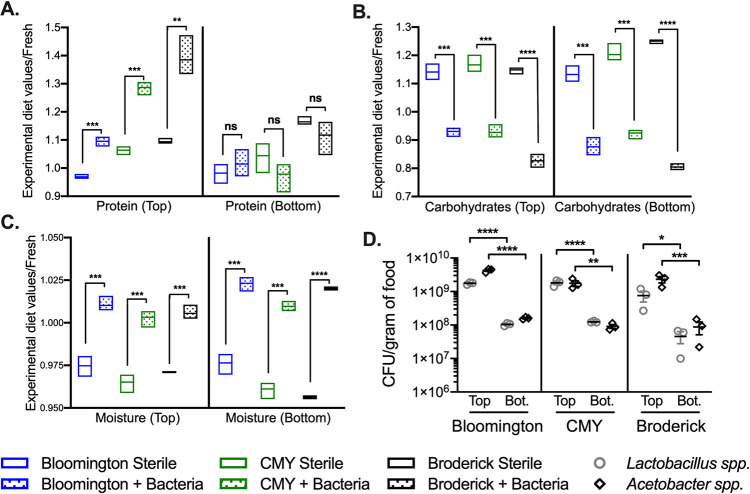
Bacterial growth on fly food results in nutrient stratification. Protein (A), carbohydrates (B), moisture (C), and bacterial load (D) in Bloomington, CMY, and Broderick diets were assessed 11 days after inoculation with sterile embryos and PBS (Sterile) or 10^4^ cells of a 4-species bacterial cocktail (+Bacteria). The top and bottom halves of food were analyzed as separate samples. Nutritional data (A to C) are expressed as raw values from each nutritional test in treated samples divided by raw values for fresh, untreated diets (see Fig. S1 in the supplemental material for raw nutritional data). Bars represent minimum and maximum values and means from 3 biological replicates. Statistical differences between sterile and +Bacteria treatments within the top or bottom of each diet were determined using unpaired two-tailed *t* tests. Bacterial loads (D) are expressed as CFU per gram of food, with each point representing an individual replicate. Lines and error bars represent means ± SEM. Statistical differences between *Lactobacillus* spp. and *Acetobacter* spp. in the top versus bottom food were determined via two-way ANOVA. Bacterial growth was not detected in sterile treatments and is not shown. Significance is expressed as follows: ns, not significant (*P* > 0.05); *, *P* ≤ 0.05; **, *P* ≤ 0.01; ***, *P* ≤ 0.001; ****, *P* ≤ 0.0001.

To account for possible differences in food layer depth, we next separately analyzed the nutritional contents of the top versus bottom layers of food after treatment with bacteria/PBS and bleached fly embryos. Carbohydrates were decreased and moisture was increased in both the top and bottom layers of food, consistent with the whole-sample analysis (Fig. 2A to C). However, in separating the top and bottom halves of the diet, we observed an increase in protein in the top, but not the bottom, layer of food (Fig. 2A), which was correlated with a 10-fold-higher bacterial density in the top than in the bottom half of food, although bacterial counts of both *Lactobacillus* spp. and *Acetobacter* spp. were substantial in both layers (Fig. 2D). Altogether, these data indicate that bacteria are capable of changing the nutritional environment of the food, resulting in relatively low carbohydrate and high moisture levels throughout, while generating a stratification of protein that correlates with bacterial density.

### Environmental moisture and dietary protein-to-carbohydrate content dictate life history traits in axenic flies.

While the increase in protein and decrease in carbohydrates due to gut bacteria were not surprising based on previous studies showing the influence of bacteria on protein nutrition and carbohydrate utilization (11, 12), we were not expecting such a significant shift in moisture content. We hypothesized that in addition to altering protein and carbohydrates, bacterial manipulation of moisture in fly diets would have resulting effects on fly life history.

**FIG 3:**
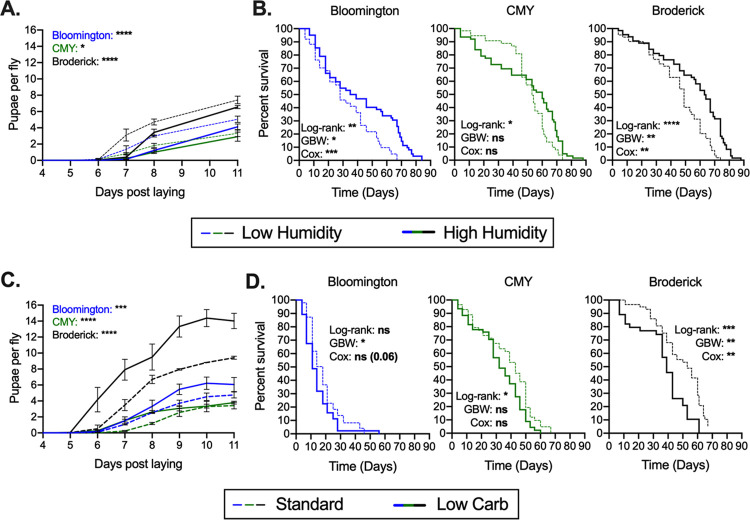
Environmental moisture and dietary protein-to-carbohydrate content dictate life history traits in axenic flies. (A and B) Fecundity (A) and longevity (B) of axenic flies reared on the Bloomington, CMY, and Broderick diets at low (27%) or high (85%) relative humidity (RH). (C and D) Fecundity (C) and longevity (D) of axenic flies reared on standard Bloomington, CMY, and Broderick diets (Standard) or with carbohydrates reduced by 29% (Bloomington), 42% (CMY), or 31% (Broderick) in an attempt to match the protein-to-carbohydrate ratio of the top half of each diet with bacteria as determined from nutritional analyses in Fig. 2 (Low Carb treatment). Each graph (A to D) represents the averages from three replicate experiments starting with 18 to 25 4-day-old adult females. Fecundity (A and C) is expressed as the number of pupae recorded for 11 days divided by the number of laying adult females in a vial. Lines represent means from replicates ± SEM. Statistical analyses were performed by two-way ANOVA with Bonferroni multiple comparisons for low- versus high-humidity treatments (A) or standard and low-carbohydrate treatments (C) within each diet. Longevity (B and D) is expressed as Kaplan-Meier survival, with statistical differences between low and high humidity (B) or between standard and low-carbohydrate treatments (D) on each diet determined via log rank, Gehan-Breslow-Wilcoxon (GBW), and Cox mixed-effects analyses for the data from 3 replicate experiments combined. Survival curves for individual replicates are shown in Fig. S2 in the supplemental material. Significance is expressed as follows: ns, not significant (*P* > 0.05); *, *P* ≤ 0.05; **, *P* ≤ 0.01; ***, *P* ≤ 0.001; ****, *P* ≤ 0.0001.

To increase moisture in fly food without diluting the diet, we reared axenic flies under conditions of low (27%) or high (85%) relative humidity (RH). Fly vials placed in high humidity were also treated with 300 μl of 1% agar as described previously by Ja et al. to further promote water availability (18). We found that high humidity delayed development and extended longevity across diets (Fig. 3A and B). These results are consistent with previous studies showing that increased dietary water content slowed larval development and lengthened the life span of flies fed a concentrated, nutrient-rich diet (18, 19). Our results showing that this is influenced by the microbiome suggest a novel mechanism by which microbial association can impact D. melanogaster physiology.

The observed changes in protein and carbohydrate levels in food with bacteria are reminiscent of dietary restriction studies in which alteration of the protein-to-carbohydrate (P:C) ratio leads to significant effects on life history (20, 21). To measure the impact of microbe-mediated dietary carbohydrate and protein changes, we prepared diets with a roughly 30% lower carbohydrate content by decreasing the amounts of corn syrup in the Bloomington diet, molasses in the CMY diet, and sucrose in the Broderick diet. Consequently, the resulting “low-carbohydrate” diets were composed of P:C ratios similar to those of the top half of each diet containing bacterial treatments from Fig. 2 (Table S1). A number of dietary restriction studies have shown that a decreased P:C ratio results in reduced fecundity and extended longevity. Here, increasing the P:C ratio through carbohydrate reduction increased fecundity with variable effects on longevity in a diet-dependent manner (Fig. 3C and D). While the low-carbohydrate version of the Broderick diet significantly decreased the life span, the differences between the standard and low-carbohydrate Bloomington and CMY diets were less conclusive, with significance metrics differing based on the statistical test applied. This variability, while likely a factor of sample size, can possibly also be attributed to the different magnitudes of changes in carbohydrate contents between the standard and low-carbohydrate treatments across all diets (based on sterile and +Bacteria nutritional analyses) (Table S1). The feeding rate was measured in young flies on each diet and was found to be unaffected by low-carbohydrate conditions on the CMY diet but was slightly increased in flies on the Bloomington and Broderick low-carbohydrate diets compared to controls (Fig. S2). We also noted that the feeding rate was noticeably lower in general on the two Bloomington diets than on the other diets, which may explain the significant amounts of early death on the Bloomington diets (Fig. 3D; Fig. S2 and S3).

### Gut bacteria increase dietary tryptophan with resulting impacts on longevity.

**FIG 4:**
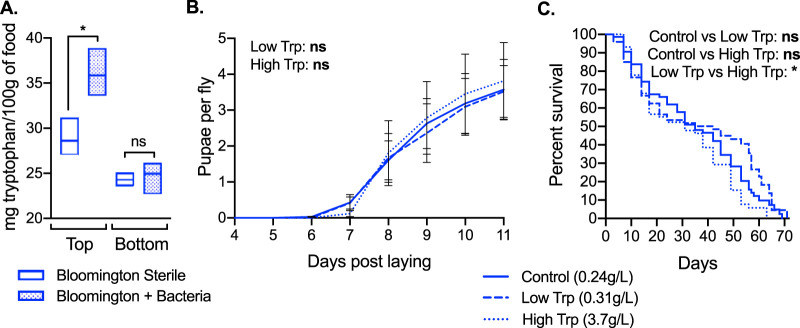
Gut bacteria increase dietary tryptophan, with resulting impacts on longevity. (A) Amount of tryptophan in the Bloomington diet 14 days after inoculation with PBS (Sterile) or 10^4^ cells of a 4-species bacterial cocktail (+Bacteria). As for Fig. 2, the top (∼7 mm) and bottom (∼7 mm) halves of food were analyzed separately. Bars represent minimum and maximum values and means from 3 biological replicates. Statistical significance of sterile versus +Bacteria treatments was determined using unpaired two-tailed *t* tests. (B and C) Fecundity (B) and longevity (C) of axenic female flies reared on the Bloomington diet prepared as standard (control) (0.24 g/liter total tryptophan), supplemented with 71.5 mg l-tryptophan (Low Trp) (0.31 g/liter total tryptophan), or supplemented with 3.5 g l-tryptophan (High Trp) (3.7 g/liter total tryptophan). Results are compiled from 3 replicate experiments starting with 25 3-day-old females each. Fecundity (B) is expressed as the number of pupae recorded for 11 days divided by the number of laying adult females in a vial. Lines represent means ± SEM. Statistical analyses were performed by two-way ANOVA with Dunnett’s multiple-comparison analysis comparing low- and high-tryptophan samples to the control. Longevity (C) is expressed as Kaplan-Meier survival, with statistical differences between each treatment determined via a Cox mixed-effects survival model. Survival curves for individual replicates are shown in Fig. S2 in the supplemental material. Significance is expressed as follows: ns, not significant (*P* > 0.05); *, *P* ≤ 0.05.

To further understand microbe-mediated impacts on protein content in the context of amino acid provisioning, we analyzed the Bloomington diet for amino acid content with and without 14 days of bacterial growth. The only amino acid significantly impacted by bacteria was tryptophan, which increased in the top half of food with bacterial inoculation (Fig. 4A; see also Fig. S4 in the supplemental material for the complete amino acid profile). We then examined whether increases in tryptophan due to bacteria in food influence host life history traits. We prepared the Bloomington diet as normal (control) (containing 0.24 g/liter tryptophan) or supplemented food with tryptophan that either matched microbe-induced levels (low Trp) (0.31 g/liter total tryptophan) or greatly increased the tryptophan concentration (high Trp) (3.7 g/liter total tryptophan) and monitored the life history of axenic flies on each treatment. We observed no impact of altered tryptophan on fly fecundity (Fig. 4B), but for longevity, we found that low and high tryptophan, while neither was statistically different from the control, were significantly different from each other, with the life span being reduced on high-tryptophan compared to low-tryptophan diets (Fig. 4C). The implications of these results are challenging to interpret, as control survival rates varied considerably across replicate experiments (Fig. S3). It is likely that the small difference in tryptophan levels between the control and the low-Trp treatment (0.24 g/liter compared to 0.31 g/liter) is not sufficient to visualize a change in life span, but it is unclear if the same can be said between control and high-Trp treatments (0.24 g/liter compared to 3.7 g/liter). Regardless, a significant reduction in life span, particularly in flies surviving longer than 30 days, was observed for high Trp compared to low Trp, suggesting a role for tryptophan in aging on the Bloomington diet.

### Gut bacteria influence host life history differentially based on fly diet.

**FIG 5:**
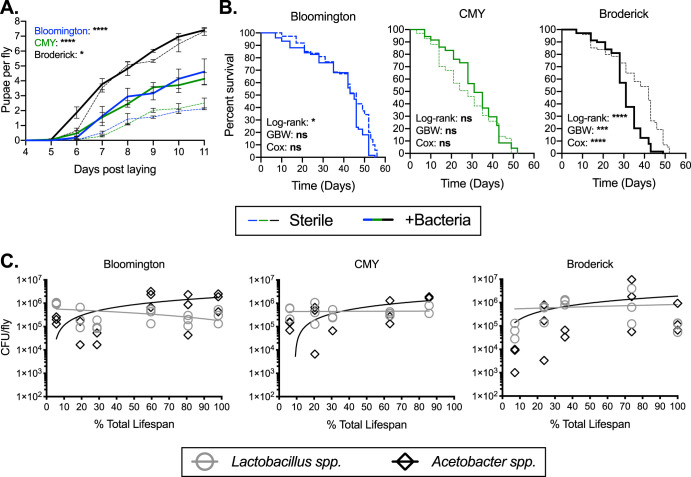
Gut bacteria differentially influence host life history based on fly diet. Fecundity (A), longevity (B), and bacterial load (C) of (initially) axenic female flies maintained on the Bloomington, CMY, and Broderick diets supplemented with PBS (Sterile) or 10^4^ bacterial cells (+Bacteria) with each passage to fresh food were determined. Fecundity, longevity, and bacterial load data were obtained from 3 (A and B) or 1 (C) replicate experiment consisting of 21 to 25 4-day-old adult flies at the start of the experiment. Fecundity (A) is expressed as the number of pupae recorded for 11 days divided by the number of laying adult females in a vial. Lines represent means from replicates ± SEM. Statistical analyses were performed by two-way ANOVA with Bonferroni multiple comparisons for sterile and +Bacteria treatments within each diet. Longevity (B) is expressed as Kaplan-Meier survival, with statistical differences between sterile and +Bacteria treatments on each diet determined via log rank, Gehan-Breslow-Wilcoxon (GBW), and Cox mixed-effects analyses for data from the 3 replicate experiments combined. Survival curves for individual replicates are shown in Fig. S3 in the supplemental material. Bacterial load (C) is shown as a combination of L. plantarum and L. brevis (*Lactobacillus* spp.) or of A. pasteurianus and A. tropicalis (*Acetobacter* spp.) for individual flies (each point = 1 fly). Best-fit lines for each genus were determined via linear regression (no significant difference in *Lactobacillus* spp. or *Acetobacter* spp. when each diet was compared with another). Significance is expressed as follows: ns, not significant (*P* > 0.05); *, *P* ≤ 0.05; ***, *P* ≤ 0.001; ****, *P* ≤ 0.0001.

To contextualize our results showing life history impacts of the P:C ratio, moisture, and tryptophan, we next addressed how microbes themselves affect fly physiology on the three standard diets. We found that diet was a crucial factor determining microbial impacts on the time to pupation, the number of pupae per fly, and the fly life span. While +Bacteria treatments profoundly increased pupa numbers on the Bloomington and CMY diets, it had no effect on total pupa counts on the Broderick diet (possibly due to a decrease in the feeding rate with bacterial treatment) (Fig. S2); however, the time to pupation was faster for all three diets with bacterial treatment (most evident at day 6 for the Broderick diet and day 7 for the Bloomington and CMY diets) (Fig. 5A). The life span of flies exposed to bacteria was most impacted on the Broderick diet, with +Bacteria conditions reducing the median life span by about 10 days compared to sterile controls (Fig. 5B). Sterile and +Bacteria Bloomington and CMY flies did not have discernably different survival rates (Fig. 5B). The feeding rate of sterile flies compared to +Bacteria treatments was slightly higher on the Bloomington and CMY diets and significantly higher on the Broderick diet (Fig. S2). No differences in bacterial load or composition (by genus) were observed in flies throughout their life span across the three diets (Fig. 5C), suggesting that diet-specific bacterial effects on fecundity and longevity were not due to differences in bacterial growth but potentially were due to microbial utilization and/or provisioning of nutrients or other metabolites that differ based on the specific dietary composition.

### Gut bacteria impact nutrition in a natural food substrate.

**FIG 6:**
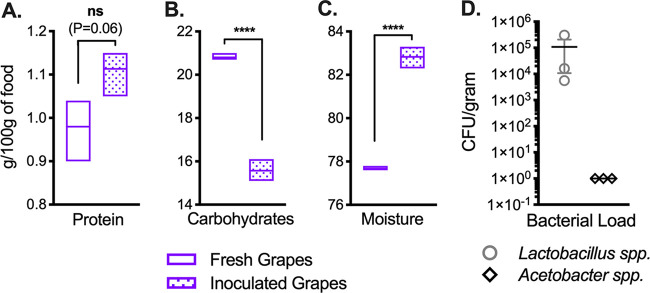
Gut bacteria impact nutrition in a natural food substrate. Protein (A), carbohydrates (B), moisture (C), and bacterial load (D) in crushed grapes either immediately after inoculation with PBS (Fresh) or 14 days after inoculation with 10^4^ cells of a 4-species bacterial cocktail (Inoculated) are shown. Bars in panels A to C represent minimum and maximum values and means from 3 biological replicates. Statistical differences between fresh and inoculated treatments were determined using unpaired two-tailed *t* tests. Bacterial loads (D) are expressed as CFU per gram of food, with each point representing an individual replicate. Lines and error bars represent means ± SEM. Bacterial growth was not detected on fresh grapes and is not shown. Significance is expressed as follows: ns, not significant (*P* > 0.05); ****, *P* ≤ 0.0001.

Having investigated microbe-mediated changes in nutrition on three standard laboratory diets, we asked how the observed nutritional changes translated to a natural fly dietary substrate. We analyzed the nutritional content of grapes as fresh samples (with PBS) or after inoculation with the 4-species bacterial cocktail after 14 days of incubation. As observed for standard diets (Fig. 1), bacterial growth on grapes resulted in a decrease in carbohydrates and an increase in moisture; a trend toward higher protein levels was also observed but did not meet significance at the level of a *P* value of 0.05 (*P* = 0.06) (Fig. 6A to C). Surprisingly, we detected only *Lactobacillus* spp. after incubation, suggesting that the observed nutritional effects, at least on grapes, were mediated primarily by *Lactobacillus* and were not dependent on coculture with or contributions from *Acetobacter* (Fig. 6D). Together, these results confirm that the nutritional relationship between D. melanogaster gut microbes and standard laboratory fly food is not only reproducible in a natural food source but also physiologically relevant.

## DISCUSSION

Gut microbes can rescue the detrimental effects of flies feeding on diets lacking protein (8, 11) yet reduce the life span under certain nutritionally rich conditions (15). However, the nutritional impact of the microbiota of fruit flies reared on “standard” laboratory diets or in natural dietary environments has not been extensively explored. In this study, we investigated the impact of a representative bacterial community on three standard diets and a natural diet of grapes on the nutritional makeup of food and demonstrate their effects on host-microbe physiology. Because of D. melanogaster’s close association with its microbe-rich food source, which heavily dictates the host’s internal microbiome (22), studies of fly diet external to the host provide insight into critical biological processes mediated by the microbiome.

### Nutrient stratification.

Growth of bacteria on each diet resulted in decreases in carbohydrates and increases in moisture throughout the food, yet protein was detectably increased only in the top half of the food. This shows that microbial growth/activity leads to nutrient heterogeneity in the fly substrate (Fig. 2). The stratification of protein is correlated with higher bacterial counts in the top half of food, suggesting that the bacterial biomass itself contributed to the significant increase in dietary protein. The fact that carbohydrate and moisture levels were constant throughout the food, unlike protein, suggests that the higher bacterial biomass on the food surface was not accompanied by increased bacterial metabolism (which would result in sugar consumption and presumably moisture production), possibly due to a “maxing out” of metabolic activity throughout food despite the varying rates of cell division (23). Protein stratification due to bacteria is important to consider in the context of nutrition-dependent development and longevity, as larvae and adults may be exposed to different nutritional environments. Larval density likely also impacts this nutrient stratification such that increased churning of the substrate at the food surface may lead to better mixing of food layers than what is seen in tubes lacking (or with fewer) larvae (24).

### Microbiome and dietary plenitude.

Dietary restriction studies have identified decreased protein availability as a factor that consistently prolongs life span and health span across model organisms (21). In flies, this effect is tied to insulin signaling and the TOR pathway, which are stimulated by both diet and the microbiome (8, 9, 25, 26). Keebaugh et al. recently showed that live and dead bacteria extended the life span of protein-deficient flies (11) but had less of an effect on flies on high-nutrient diets (15). These studies identify protein availability as a major factor defining the host-microbe relationship but acknowledge that the overall impacts of microbes on life span are likely a combination of nutritional, host, and microbial factors. We show that the microbiome itself alters the nutritional composition of the diet to generate conditions of dietary plenitude, specifically an excess of protein accompanied by decreased carbohydrates, and that this occurs to similar extents on three standard diets despite different nutritional compositions. While these dietary changes result in developmental and longevity phenotypes characteristic of both flies on high-P:C-ratio diets and flies reared with microbes on the Broderick diet, the relationship is not as obvious with regard to longevity on the Bloomington and CMY diets. Life span differences between sterile and +Bacteria treatments on Bloomington and CMY food were generally nonsignificant, suggesting further nutrient-mediated effects of bacteria on life history independent of protein and carbohydrates. In interpreting our longevity data, it is important to acknowledge that the frequency of transfer used in our analysis is lower (every 3 to 4 days) than that commonly used in life span assays (every day). It is interesting to note that more frequent (daily) transfers of flies are associated with reduced microbiome density (15, 27–29). Thus, it is possible that much of the life span literature is reporting on effects that are largely independent of the microbiome or at least in flies with depleted microbiomes. Such impacts on the microbiome could also account for differences across studies, and it would be useful to take this into consideration in future studies and interpretation of published data.

Regarding the observed differences in bacterial impacts on life history between diets, one possible factor involved is micronutrients, which differ between diets based on specific nutrient compositions (17, 30–33). While we did not directly test the effect of micronutrient differences among the three standard diets, we can make some predictions based on their unique dietary components. For example, the CMY diet contains molasses, which is rich in choline (13.3 mg per 100 g of molasses [17]). Choline is a compound known both to be metabolized by gut bacteria (34) and to impact D. melanogaster growth and development (35), making it just one of many possible candidate micronutrients that could conceivably modulate microbiome-diet-host relationships.

It is also prudent to consider the importance of links between feeding behavior and dietary conditions with regard to life history. We observed a trend of increased feeding rates in flies fed a low-carbohydrate (high-P:C-ratio) diet (see Fig. S2 in the supplemental material), with these flies exhibiting a tendency toward shorter life spans than controls (Fig. 3D), potentially suggesting that increased consumption of food/protein along with the already altered diet may be a factor dictating life span effects. However, flies reared with bacteria generally had lower feeding rates than sterile controls (Fig. S2), with survival being either decreased (Broderick diet) or unchanged (Bloomington and CMY diets) (Fig. 5), meaning that feeding behavior purely in response to protein/carbohydrates differs from feeding behavior in response to bacteria. This is unsurprising as it is known that fly-associated microbes produce metabolites that impact fly behavior (14), but future investigations into how the protein in bacteria contributes to these feeding effects will be of great interest.

### The microbiome as a regulator of tryptophan metabolism.

In addition to micronutrients, amino acids are likely to impact host biology in different ways based on host-microbe, host-diet, and microbe-diet relationships (10, 36–40). Our analysis showed that tryptophan was significantly increased with bacterial growth and, when administered in the diet to axenic flies, impacted fly life span. As a precursor for the hormone serotonin, tryptophan’s connection with the microbiome is the subject of many recent investigations of the gut-brain axis, that is, the role of the microbiome in cognitive function and as a factor in disorders such as anxiety and depression (reviewed in references 41 and 42). Studies in flies and rodents exploring the function of tryptophan in host physiology independent of the microbiome have identified a role for the amino acid in aging-related pathologies, some even drawing links between tryptophan deficiency and dietary restriction (43–48). As for humans, tryptophan is an essential amino acid for D. melanogaster, meaning that the amino acid must be acquired from dietary sources for complete nutrition (49). Yet tryptophan is relatively scarce in nature and metabolically expensive for plants and microbes to produce (50), highlighting microbiome-mediated tryptophan metabolism as an important aspect of D. melanogaster host-microbe physiology. It is unclear if any of the gut microbes utilized in this study biosynthesize tryptophan or if it is extracted from dietary proteins but not utilized (39, 51, 52), so additional work exploring microbial regulation of tryptophan in D. melanogaster will be of great interest.

### Microbe-derived moisture.

Our study links the growth of gut bacteria on the fly diet to significant increases in dietary moisture, but it is not yet clear how such changes contribute to microbe-mediated host physiology. Our results suggest that the relationship between bacteria, dietary moisture, and life history is complex, as we observed the microbiome as a factor reducing the development time and shortening the life span on two of three diets, contrary to what we observed with increased environmental moisture. It is likely that factors modulating the impact of the microbiome on life history are multifaceted, and microbial contributions to dietary moisture may not be significant enough *in vivo* to greatly affect physiology.

With regard to behavioral physiology, increased moisture likely contributes to the attractiveness of fermented foods to the fly. “*Drosophila*,” derived from Greek, means “dew-loving,” indicating a long-standing appreciation for the necessity of moist environments for fly habitation (53). Sayeed and Benzer reported hygrosensory behavior in D. melanogaster, confirming that moisture is specifically sought out by the fly (54). Previous work investigating attractive compounds produced by gut microbes focused primarily on metabolites and odorants (14, 55–57), but the microbiome has been underappreciated as a source of attractive moisture content in the dietary substrate. It is also not clear how the microbiome increases food moisture content, but it is possible that this could be directly as a by-product of metabolism or indirectly through reduced evaporation from the food surface, for example, via biofilms or surfactants (58, 59).

### Physiological relevance.

We conclude our study by confirming that the protein, carbohydrate, and moisture changes in standard fly diets translate to a natural fly dietary substrate inoculated with bacteria. Because we also confirmed that inoculating standard diets via gnotobiotic flies results in nutritional changes similar to those with culture inoculation, we expect that the observed nutritional changes occur in fly-associated food substrates in nature. It has long been appreciated that microbes, including *Lactobacillus*, *Acetobacter*, and yeasts, are crucial for promoting fly associations with natural dietary sources, including fruit (reviewed extensively in reference 60). Our study suggests that in modifying the substrate to provide a plentiful, moist, nutritional environment, D. melanogaster-associated microbes create an attractive, hospitable niche supportive of larval development and adult homeostasis. Future work exploring whether micronutrient differences in diet or specific microbiome compositions vary microbe-mediated effects on the host will greatly expand our understanding of fly nutritional physiology and ecology.

## MATERIALS AND METHODS

### Fly diets.

Diets used for nutritional analyses included the Bloomington standard Nutri-Fly formulation (catalog number 66-113; Genesee Scientific), the Bloomington molasses Nutri-Fly formulation (catalog number 66-116; Genesee Scientific), and the Broderick standard diet (Table 1). For life history experiments with altered carbohydrates, Bloomington standard, Bloomington cornmeal-molasses-yeast, and Broderick standard diets were made from scratch with reduced amounts of corn syrup, molasses, and sucrose, respectively (Table 1). Life history experiments with diets containing an altered tryptophan content were done using the Bloomington standard Nutri-Fly formulation with 71.5 mg (low) or 3.5 g (high) of l-tryptophan (catalog number T8941; Sigma) (added as described in reference 48). Fly food was prepared by boiling 1 liter of distilled water, stirring-in dry ingredients, and allowing food to cook for 20 min. Methyl paraben was added last before mixing food via an immersion blender. Diets were transferred to wide vials (10 ml) or 6-oz bottles (50 ml). Vials/bottles were plugged and autoclaved for 20 min at 121°C.

**TABLE 1:** Description of experimental diets

Diet	Source and/or components per 1 liter of water
Bloomington standard (Nutri-Fly)	Genesee Scientific (catalog no. 66-113) (Nutri-Fly BF): yellow cornmeal, agar (type II), corn syrup solids, inactive nutritional yeast, soy flour, 4.4 ml propionic acid added during cooking
Bloomington molasses (CMY) (Nutri-Fly)	Genesee Scientific (catalog no. 66-116) (Nutri-Fly MF): cornmeal, agar (type II), molasses solids, inactive nutritional yeast, 1.4 g methyl paraben in 10 ml 100% ethanol added during cooking
Broderick standard	50 g inactive dry yeast, 70 g yellow cornmeal, 6 g agar, 40 g sucrose, 1.25 g methyl paraben in 5 ml 100% ethanol
Bloomington standard (homemade)	15.9 g inactive dry yeast, 67 g yellow cornmeal, 9.2 g soy flour, 5.29 g agar, 102 ml corn syrup, 4.4 ml propionic acid^*a*^ (recipe makes 42.5 liters, so all ingredient amts were divided by 42.5 to make 1 liter)
Bloomington CMY (homemade)	12.4 g inactive dry yeast, 61.3 g yellow cornmeal, 6.01 g agar, 75.2 ml molasses, 1.4 g methyl paraben in 10 ml 100% ethanol^*b*^ (for this recipe, values were divided by 2.66 to make 1 liter)
Bloomington low carbohydrate	15.9 g inactive dry yeast, 67 g yellow cornmeal, 9.2 g soy flour, 5.29 g agar, 41 ml corn syrup, 4.4 ml propionic acid
CMY low carbohydrate	12.4 g inactive dry yeast, 61.3 g yellow cornmeal, 6.01 g agar, 15 ml molasses, 1.4 g methyl paraben in 10 ml 100% ethanol
Broderick low carbohydrate	50 g inactive dry yeast, 70 g yellow cornmeal, 6 g agar, 0 g sucrose, 1.25 g methyl paraben in 5 ml 100% ethanol

a See https://bdsc.indiana.edu/information/recipes/bloomfood.html.

b See https://bdsc.indiana.edu/information/recipes/molassesfood.html.

### Preparation of the inoculum.

Lactobacillus plantarum, Lactobacillus brevis, and Acetobacter pasteurianus strains used were previously isolated from laboratory-reared Drosophila melanogaster (28). Acetobacter tropicalis DmCS_006 was obtained from John Chaston (37). Liquid cultures were prepared as follows, with shaking at 200 rpm: *Acetobacter* strains were grown for 1 day in a 1:1 mixture of de Man, Rogosa, and Sharpe (MRS) broth plus mannitol, and *Lactobacillus* strains were grown for 1 day in MRS broth. Cultures were centrifuged for 20 min at 4°C at 3,428 × *g*, the supernatant was removed, and cells were resuspended in sterile PBS. Each bacterial culture was then quantified using a hemocytometer and set to a concentration of 10^4^ cells per 500 μl in PBS. Each strain was then combined in a 1:1:1:1 mixture, and 500 μl of the bacterial mix were pipetted onto fly food for nutritional analysis experiments; sterile controls were treated with 500 μl of PBS.

### Axenic and gnotobiotic flies.

All flies used in this study were from the Oregon-R background (which contains *Wolbachia*) from the Bloomington *Drosophila* Stock Center (Bloomington, IN). Flies were maintained in a 25°C 12-h-light–12-h-dark incubator in ambient humidity (around 27% RH). Axenic flies were generated by letting flies lay eggs overnight on grape juice agar plates smeared with yeast paste, scraping eggs into cell strainer baskets using swabs, and applying 10% bleach. Once the chorion was visually confirmed to have disappeared, eggs were washed with sterile water and ethanol before being transferred via a pipette to sterile fly food for development. The bacterial inoculum was prepared as described above, and 150 μl of the 4-species cocktail was fed to axenic adults for 4 days (one initial inoculum) before 20 male and 25 female flies were used to inoculate experimental diets for nutritional tests. At the time of inoculation via gnotobiotic flies, subsets of flies were homogenized and plated on MRS agar to determine *in vivo* bacterial counts.

### Nutritional analyses.

Experimental diets in 6-oz fly bottles were treated as follows and incubated at 25°C for the specified time periods. For culture inoculation for whole-sample analysis, diets were inoculated directly with PBS or bacteria and incubated for 14 days (Fig. 1A to D; see also Fig. S5A in the supplemental material). For gnotobiotic inoculation for whole-sample analysis, diets were subjected to axenic (control) or gnotobiotic flies for 4 days and incubated without flies for an additional 10 days (Fig. 1E to H; Fig. S5B). For culture and embryo inoculation for top-versus-bottom analysis, diets were treated with 32 μl sterile embryos (as described in reference 61) and direct inoculation of PBS/bacteria, with 11 days of incubation (Fig. 2; Fig S5C). For culture inoculation for top-versus-bottom amino acid analysis, diets were inoculated directly with PBS or bacteria and incubated for 14 days (Fig. 4; Fig. S5D). For top-versus-bottom experiments, the food line was measured after the incubation period, the bottle was cut open using a sterile razor, and the top and bottom halves of the food were collected separately using autoclaved weigh boats. Fresh red grapes were washed with water and crushed with a sterile gloved hand, and 50 g was distributed into sterile beakers. Grapes were inoculated with 500 μl of PBS (fresh) or the 4-species cocktail (inoculated) as shown in Fig. S5A in the supplemental material, covered with sterile aluminum foil, and collected either immediately (fresh) or after 14 days of incubation at 25°C (inoculated).

Food samples were collected into 50-ml conical tubes and analyzed by Eurofins Food Integrity & Innovations (Madison, WI) for protein, carbohydrates, fat, ash, moisture, caloric content, and, in one experiment, amino acids.

At the time of food collection postincubation, 0.5 g of food (mixed well in a conical tube) was collected and homogenized in PBS, serially diluted, and plated on MRS agar to quantify *Lactobacillus* and *Acetobacter* by genus.

### Fecundity and life span experiments.

Axenic flies were generated as described above and maintained on the Broderick diet. Laying adults (F0) were transferred to the test diets at least 2 weeks prior to beginning longevity and fecundity analyses, ensuring that the progeny (F1 and later) used for experiments had developed in full on the test diet. On experimental day 0, 3- to 4-day-old adult females (18 to 25 flies per vial) were collected in a biosafety cabinet with laminar flow in order to maintain sterility. Vials were flipped twice per week for the duration of the fly life span (11, 15). Fecundity was recorded as the number of pupae that developed each day for the first 11 days following the start of the experiment. Longevity was recorded as the number of dead flies twice per week at the start of the experiment and more frequently toward the end of the fly life span. Flies that died due to being stuck in the food were censored. Vials were kept at 25°C with ambient (∼27%) relative humidity except for the high-humidity test group, which was kept in a 25°C 12-h-light–12-h-dark incubator set to 85% RH. Vials placed in high humidity were also supplemented with 300 μl of 1% agar on the side of the tube, as described previously (18).

### Feeding score.

Axenic flies that were adapted to experimental diets were placed on appropriate diets (without starving) in which 0.1% erioglaucine disodium salt (final concentration in food) had been thoroughly incorporated and treated as described above for each test (i.e., inoculated with PBS, bacteria, or nothing). Ten to 20 female flies (3 to 4 days old) fed for 2 h at 25°C at the same time of day (12:00 p.m. to 2:00 p.m.) for each replicate experiment and guts from 5 randomly chosen flies per replicate per treatment were dissected. Qualitative feeding scores were assigned to dissected guts by multiplying the intensity of blue coloring (determined visually) by the percentage of the gut exhibiting blue color.

### Data analysis and statistical tests.

*t* tests comparing sterile and +Bacteria-treated food were performed using R. Two-way analyses of variance (ANOVAs) with Bonferroni multiple comparisons were performed for bacterial load analyses and pupa counts using GraphPad Prism. Feeding scores were analyzed in GraphPad Prism using Anderson-Darling normality tests followed by one-tailed Wilcoxon tests using the Pratt method. Differences in survival between treatments on each diet were analyzed using Kaplan-Meier survival curves and log rank and Gehan-Breslow-Wilcoxon (GBW) statistical tests in GraphPad Prism; these statistical tests were used to show overall survival differences (log rank) and differences driven by early death (as GBW analysis weights early time points more heavily than late ones). Additionally, survival differences between treatments were also analyzed via a Cox mixed-effects survival model with Weibull distribution using Stata, accounting for random effects of replicates. Linear regression comparing bacterial loads over the course of the fly life span was performed in GraphPad Prism. Significance is expressed as indicated in the figure legends.

## Supplementary Material

Supplemental file 1
